# Development of healthier gummy candy by substituting glucose syrup with various fruit juice concentrates

**DOI:** 10.1002/fsn3.4389

**Published:** 2024-08-13

**Authors:** Serpil Pekdogan Goztok, Arezou Habibzadeh Khiabani, Omer Said Toker, Ibrahim Palabiyik, Nevzat Konar

**Affiliations:** ^1^ Department of Food Technology, Vocational School of Technical Sciences Siirt University Siirt Turkey; ^2^ Food Engineering Department, Chemical and Metallurgical Engineering Faculty Yildiz Technical University İstanbul Turkey; ^3^ Food Engineering Department, Agricultural Faculty Namik Kemal University Tekirdağ Turkey; ^4^ Department of Dairy Technology, Faculty of Agriculture Ankara University Ankara Turkey

**Keywords:** confectionery, fruit juice concentrate, glucose syrup, healthy candy, natural sweeteners

## Abstract

The main objective of this study was to evaluate the effects of various formulations of fruit juice concentrates (pomegranate, grape, and sour cherry) on the pH, water activity, density, color, texture, and microstructure characteristics of candies instead of glucose syrup. The experimental points of the study were examined by a D‐optimal mixture design to optimize the concentration of fruits used in the formulation and achieve excellent physicochemical characteristics. Fruit juice concentrates, either singly or in combination, were used as a complete substitute for glucose syrup in the formulation. Total fruit juice concentration used in the formulation was 54.07% and each of the fruit juice concentrations changed between 0 and 54.07% in the formulation. By combining these three fruit juices, 14 gummy candy samples were produced, depending on the Special cubic, cubic, and quadratic models that were used for the effects on the physicochemical properties (pH, water activity, density, L*, a*, b*, and chroma), and the texture profile analysis (TPA) (hardness, cohesiveness, springiness, and resilience) parameters according to independent variables. Results showed that pH, water activity, and density values of the gummy candy samples were found to be in the range of 2.22–3.08, 0.46–0.52, and 1.10–1.53 g/mL, respectively, and were significantly affected by different fruit juice concentrates (*p* < .05). The texture profile analysis showed that except for springiness, fruit juice concentrations significantly affected the texture profile (*p* < .05). The texture values, such as hardness, springiness, cohesiveness, gumminess, and resilience of the gummy candy samples, were determined as 146.1–938.8 N, 0.63–0.99, 0.75–1.19, 136.02–947.94 g, and 0.12–0.51, respectively. In addition, various fruit juice concentrates significantly affected the color parameters of gummy candies, and using pomegranate juice and sour cherry concentrates increased the +a* value of the gummy candies. Therefore, fruit juice‐based gummy candies can be developed as value‐added gummy candies by using fruit juice concentrates.

## Graphical abstract

The objectives of this study were to investigate the effects of using various combinations of fruit juice concentrates (pomegranate, grape, and sour cherry) on the physicochemical (pH, water activity, density, and color), texture, and microstructure properties of gummy candies instead of glucose syrup.

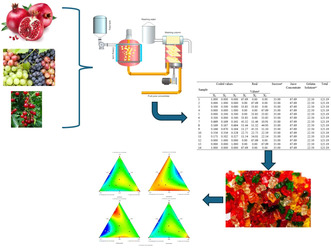

## 1 INTRODUCTION

One of the main components of confectionery products is candies such as jellies, gummy candy, chewing gums, marshmallows, etc., which cater to a wide range of consumers, making them the most popular confections by many people, especially children (Csima et al., 2010; Solangi et al., 2022). Gummy candy and gummy jellies elastic nature can make them the most versatile confection products with different shapes, tastes, and odors (Gamal et al., 2022; Habilla et al., 2011). The gummy candy is composed of water, sweeteners such as sucrose and glucose syrup, a gelling agent (agar‐agar, pectin, starch, and gelatin), flavorings (citric acid and malic acid), and colorings (Burey et al., 2009; Kurt et al., 2022). High amounts of sweetening agents in candy could attract consumers’ attention and impact the texture properties of the product (Gan et al., 2022; Marfil et al., 2012). However, concerns about sugar content, especially corn syrups (e.g., glucose syrup), have led to exploring alternative ingredients. It has been reported that excessive consumption of sweeteners causes various health issues, including diabetes, obesity, high blood pressure, cardiovascular disease, and cancer (Bouphun et al., 2023; Gan et al., 2022; Pattarathitiwat, 2020). Consequently, the search for healthier alternatives has encouraged researchers and candy manufacturers to improve the candy formulations with natural sweeteners (fruit juice) to control the sweetener level (Amjadi et al., 2018; Gok et al., 2020). Many researchers have conducted studies on replacing sucrose and glucose syrup, such as Gok et al. (2020) studied bulking agents in gummy candies instead of glucose syrup, Pekdogan Goztok et al. (2022) studied different concentrations of fruit juice as potential replacements for corn syrup in marshmallow, Kurt et al. (2022) utilized natural sugars instead of sugar syrup in gummy candy, and Bouphun et al. (2023); Gok et al. (2020); Pattarathitiwat (2020); Periche et al. (2014) used polyols to replace sucrose in low‐sugar gummies. The most common glucose syrups in the gummy and jelly candy formulations are 63 DE (dextrose equivalent) and 42 DE (Burey et al., 2009; Efe & Dawson, 2022; Tireki et al., 2021). Sucrose plays a crucial role in gummy candy formulations to determine the texture, sensory characteristics, thermal stability, mouthfeel, and gel structure (Holm et al., 2009; Tireki et al., 2021). It also contributes to the overall mass of the candy, acting as a bulking agent and affecting the candy's density (Pekdogan Goztok et al., 2022). Additionally, sucrose improves the solubility of other ingredients, retards the crystallization, and lowers the water activity, which extends the shelf life of the candy product (Ergun et al., 2010; Hinkova et al., 2015). Glucose syrup improves sucrose solubility and retards the crystallization of sucrose, as well as lowers the water activity and extends the shelf life of the candy product (Porayanee et al., 2015). Additionally, sugar in the candy's formulation may act as a dehydrating agent by absorbing water molecules and increasing the Brix (Pekdogan Goztok et al., 2022). So as the positive aspects of sugar in candy formulation are mentioned, the complete replacement of sucrose with fruit juice concentrate could alter the product's physicochemical properties, such as texture, density, shelf life, overall sensory attributes, and water activity. Therefore, while the study aims to explore the potential of fruit juice concentrates as natural sweeteners, the inclusion of sucrose in the gummy candy formulation is necessary to maintain its desired characteristics and quality. However, there are limited reports on using healthier natural sugars (fruit‐based) to improve gummy candy's nutritional and functional properties and maintain physicochemical properties like sugar candy. Pekdogan Goztok et al., 2022 formulated various soft fruit‐origin candies instead of corn syrup. They concluded that marshmallows based on a mixture of fruit concentrates with various sugar profiles have lower water activity and pH values and improve texture properties (cohesiveness and resilience) through complex polyphenol–gelatin interactions. In this study, the optimal formulation of different mixtures of fruit juice concentration was determined with the help of a D‐optimal mixture design. It is a practical technique for identifying optimum concentrations and determining the best combination of ingredients and components to achieve the desired formulation. In recent years, it has been successfully applied by several researchers (Gok et al., 2020; Homayouni Rad et al., 2019; Pekdogan Goztok et al., 2022) to find the most cost‐effective and efficient formulations in chocolate and candy formulations. For this aim, gummy candy formulations are prepared with different fruit juice concentrations (grape, pomegranate, and sour cherry) using a D‐optimal mixture design. The effect of obtained concentrations on the physicochemical characteristics and product acceptance was investigated.

## 2 MATERIALS AND METHODS

### 2.1 Materials

Gelatin Powder 200 Bloom (Burjeel, Bursa, Turkey), sucrose (Konya Sugar, Konya, Turkey), pomegranate juice concentrate (PJC), sour cherry juice (SJC) concentrate, and grape juice concentrate (GJC) (Döhler, Karaman, Turkey) were utilized as ingredients in the formulation of gummy candy samples.

### 2.2 Experimental design and model fitting

In the conventional formulation of gummy candy, 54.07% glucose syrup is used. For this aim, the glucose syrup was completely replaced with sour cherry, pomegranate, and grape juice concentrates. The experimental design for the gummy candy formulation was determined through Design‐Expert (Stat‐Ease Inc., version 7.0, Minneapolis, USA) software. A D‐optimal design was utilized with the three independent variables such as SJC (X_1_), PJC (X_2_), and GJC (X_3_), as expressed in Table 1. Fruit juice concentrates, either singly or in combination, were used as a complete substitute for glucose syrup in the formulation. Total fruit juice concentration used in the formulation was 54.07% and each of the fruit juice concentrations changed between 0% and 54.07% in the formulation. The Design‐Expert software designed 14 trials, of which 11 were different and 3 were replicated (trials 1, 2, and 4 had one replicate). Table 1 shows the formulation of gummy candies determined from the experimental design. According to the D‐optimal approach, the effect of each concentration on the physicochemical, color, and texture characteristics was investigated, and the optimum formulation was determined. For optimization, the best responses were determined based on each concentration's impact.

**TABLE 1:** D‐optimal mixture design study model and sample formulations.

Sample	Coded values			Real values^a^			Sucrose^a^	Juice concentrate^b^	Gelatin solution^a^, ^c^	Total
	X_1_	X_2_	X_3_	X_1_	X_2_	X_3_				
1	1.000	0.000	0.000	67.69	0.00	0.00	35.00	67.69	22.50	125.19
2	0.000	1.000	0.000	0.00	67.69	0.00	35.00	67.69	22.50	125.19
3	0.500	0.500	0.000	33.85	33.85	0.00	35.00	67.69	22.50	125.19
4	0.000	0.000	1.000	0.00	0.00	67.69	35.00	67.69	22.50	125.19
5	0.000	0.500	0.500	0.00	33.85	33.85	35.00	67.69	22.50	125.19
6	0.500	0.000	0.500	33.85	0.00	33.85	35.00	67.69	22.50	125.19
7	0.669	0.169	0.161	45.32	11.46	10.91	35.00	67.69	22.50	125.19
8	0.169	0.167	0.664	11.44	11.32	44.93	35.00	67.69	22.50	125.19
9	0.166	0.670	0.164	11.27	45.33	11.10	35.00	67.69	22.50	125.19
10	0.336	0.336	0.328	22.75	22.75	22.19	35.00	67.69	22.50	125.19
11	0.171	0.502	0.327	11.54	34.01	22.14	35.00	67.69	22.50	125.19
12	0.000	1.000	0.000	0.00	67.69	0.00	35.00	67.69	22.50	125.19
13	0.000	0.000	1.000	0.00	0.00	67.69	35.00	67.69	22.50	125.19
14	1.000	0.000	0.000	67.69	0.00	0.00	35.00	67.69	22.50	125.19

*Note*: X_1_, sour cherry juice concentrate; X_2_, pomegranate juice concentrate; X_3_, grape juice concentrate.

^a^ g/100 g.

^b^ Based each concentrate's total soluble solid content values.

^c^ g, gelatin:water (1:2).

### 2.3 Sample preparation

Pekdogan Goztok et al. (2022) method was performed with a slight modification. Gummy candies were manufactured with various combinations of fruit juice concentrate using the D‐optimal mixture design (Table 1). First, various concentrations of sucrose and fruit juice were dissolved in hot water with constant stirring through a thermal mixer (Thermomix TM5, Vorwerk, Wuppertal, Germany) at 60°C, at 200–300 rpm (revolutions per minute) until a transparent mixture appeared, and°Brix measurements were performed till the temperature reached 90 ± 1 by adding a portion of water. The mixture was cooled to 60°C, after which gelatin solution (gelatin:water 1:2) was added (60°C) into the previously prepared sucrose syrup solution and kept mixing for 10 min at 200–300 rpm. Then, the prepared mixture was poured into silicone containers. Finally, the products have cooled and are stored in bags at +4°C. A control sample containing glucose syrup instead of fruit juice concentrate(s) was prepared as described above.

### 2.4 pH, water activity, and density value measurements

The samples’ water activity (aw) and density were determined according to the method described by Pekdogan Goztok et al. (2022). The pH values of gummy candies were determined using a pH meter (pH‐Meter E520, Metrohm Herisau, Switzerland) (Mutlu et al., 2018). Density values of the samples were calculated using by dividing the weight of the sample to volume and the results were given as grams per milliliter (g/mL).

### 2.5 Color measurement

The surface color of the gummy candies (L*: brightness, a*: ±red–green, and b*: ±yellow–blue) was calculated using a colorimeter (Chroma Meter CR‐400, Konica Minolta, Japan). Furthermore, chroma (C*) and hue angle (*h*°) values were determined using Equations 1 and 2 (Moghaddas Kia et al., 2020);

(1) $$C^{*}=\sqrt{a{*}^{2}+b{*}^{2}}$$

(2) $$h^{{}^{\circ}}=\arctan\;\left(b^{*}/a^{*}\right)$$

### 2.6 Textural properties

Texture analysis was performed to determine compression measurements. XT Plus Texture Analyser (Stable Micro Systems, Godalming, UK), fitted with a 35‐mm diameter cylinder probe with a load cell of 5 kg at a constant speed of 1 mm/s, was used. The time between the two compressions (50%) applied to the samples was set to 15 s until deformation was 50% of the initial height of the samples. Textural characteristics were determined by the force–time curve (Cano‐Lamadrid et al., 2020).

### 2.7 Microstructural analysis

Following the method of Chen et al. (2015), the microstructure of the gummy candies was characterized using a polarized light microscope (PLM) (Leica Microsystems, Wetzlar, Germany, and Switzerland) coupled with a polarizer at 4 × 10 × magnification. Image acquisition was performed with QCapture software (QCapture Pro 5.1, QImaging, Surrey, BC, Canada).

### 2.8 Statistical analysis

Design‐Expert program (7.0.0 trial version; Stat‐Ease Inc., Minneapolis, USA) was used to predict equations, analyze variance, and fit the models and graphical representations (contour plots). Regression coefficients of linear, quartic, quadratic, cubic, and interaction terms were determined (*p* > .05) and considered statistically significant at *p <* .05.

## 3 RESULTS AND DISCUSSION

### 3.1 pH, water activity, and density value of the samples

pH value is critical for gelation and gel stabilization since gel‐forming agents form gel at isotonic points in confectionery production (Edwards, 2000). The pH of gummy candies prepared with various fruit juice concentrates was found to be in the range of 2.22–3.08 (Table 2). This value was significantly affected by mixing of various fruit juice concentrates (*p* < .05). Samples produced only from pomegranate juice concentrates showed the lowest pH value of 2.22. In contrast, the highest pH value was the combination of fruit juice concentrates with trial 9 (11.2 CJC, 45.33 PJC, and 11.10 GJC), with a value of 3.08. The pH value of the gummy candy using glucose syrup was determined to be 2.94. The three fruit juice concentrates in formulating gummy candies led to a lower pH. Enriched gummy candy samples with pineapple and papaya caused a decrease in pH value (Romo‐Zamorrón et al., 2019), while the addition of palmyra palm caused the pH value to increase (Sumonsiri et al., 2021). According to former studies, the pH values of jelly samples fabricated with different ingredients were 3.22–3.25 (Sumonsiri et al., 2021), 3.0–3.1 (Çoban et al., 2021), and the pH of commercial pomegranate juice jelly samples was 3.89 (Ventura et al., 2013). Therefore, the types and amounts of juice added are important. Because the pH value affects the gelling power, a low pH value is desired to ensure the stability of color components such as anthocyanin. Red/garnet color, which is associated with anthocyanins, is attractive to consumers, and color preservation during the jelly process is crucial (Cano‐Lamadrid et al., 2020). Accordingly, the pH value results in our study are more appropriate for the durability of these nutritionally colorful ingredients in fruit juice concentrates. The model (Table 2) showed that the various fruit juice mixtures had a significant (*p* < .05) effect on pH values, and the *R* ^2^ value of this quadratic model was found to be 0.9401 (Table 3). According to the previous studies, the pH values of marshmallows prepared with various fruit juices were calculated as 2.54–3.21 (Pekdogan Goztok et al., 2022) and 3.35–3.39 for jelly candy (Khouryieh et al., 2005). An important factor influencing the Maillard reaction is pH. In general, increasing the pH accelerates the rate and amount of browning. The pH highly influences the reaction between sugar and amino group. Sugar's open chain form and the amino group's unprotonated form are favored at higher pH. The rate and extent of browning is a minimum at pH 3 (Martins et al., 2000). A lower pH results in an equilibrium with a greater concentration of protonated amino groups, which makes them less reactive with sugar. In addition, the color of sucrose‐based candies depends on the sugar inversion level and the syrup's pH value. The rate of reaction increases with decreasing pH (Davies & Labuza, 2000). So, according to our results, low pH greatly slows down the Maillard reaction and protects the nutritional value of gummy candies.

**TABLE 2:** Physicochemical (water activity, moisture, and density) and fitted model statistics of gummy candies.

Sample	pH	Water activity (aw)	Density (g/mL)
1	2.55 ± 0.01	0.46 ± 0.00	1.27 ± 0.13
2	2.22 ± 0.01	0.50 ± 0.00	1.10 ± 0.05
3	2.43 ± 0.02	0.50 ± 0.00	1.35 ± 0.19
4	2.95 ± 0.01	0.48 ± 0.00	1.35 ± 0.07
5	2.55 ± 0.03	0.51 ± 0.00	1.47 ± 0.08
6	2.70 ± 0.02	0.50 ± 0.00	1.27 ± 0.07
7	2.53 ± 0.03	0.50 ± 0.00	1.36 ± 0.06
8	2.47 ± 0.01	0.51 ± 0.01	1.33 ± 0.14
9	3.08 ± 0.01	0.52 ± 0.01	1.47 ± 0.15
10	2.55 ± 0.01	0.51 ± 0.01	1.41 ± 0.06
11	2.38 ± 0.02	0.51 ± 0.01	1.53 ± 0.15
12	2.23 ± 0.02	0.51 ± 0.00	1.16 ± 0.08
13	2.97 ± 0.02	0.50 ± 0.00	1.37 ± 0.16
14	2.57 ± 0.01	0.48 ± 0.01	1.18 ± 0.08
Model	Quadratic	Quadratic	Quadratic
*R* ^2^	0.9401	0.8413	0.8533
Adjusted‐*R* ^2^	0.8053	0.7422	0.7616
Predicted‐*R* ^2^	−67.07	0.3865	0.5263
Adeq, precision	8.335	8.4405	9.0572
*F*‐value	6.98	8.48	9.31
*p*‐value	0.0385*	0.0047*	0.0035*
Lack of fit	0.0007**	0.9709**	0.2694**

* Significant *p* < .05.

** Not significant *p* > .05, mean ± SD.

**TABLE 3:** The fitted models for physicochemical, color, and texture parameters of gummy samples.

Parameter	Model equation
pH	= (2.55934 × X_1_) + (2.22287 × X_2_) + (2.95870 × X_3_) + (0.194592 × X_1_ × X_2_) − (0.166578 × X_1_ × X_2_) − (0.369303 × X_2_ × X_3_) − (2.92796 × X_1_ × X_2_ × X_3_) + (15.83161 × X_1_ × X_2_ × (X_1_‐X_2_)^2^) − (15.86340 × X_1_ × X_3_ × (X_1_‐X_3_)^2^) + (9.36994 × X_2_ × X_3_ × (X_2_‐X_3_)^2^)
Water activity (aw)	= (0.46674 × X_1_) + (0.506365 × X_2_) + (0.49204 × X_3_) + (0.0659583 × X_1_ × X_2_) + (0.091084 × X_1_ × X_3_) + (0.062132 × X_2_ × X_3_)
Density (g/mL)	=(1.22746 × X_1_) + (1.14633 × X_2_) + (1.34519 × X_3_) + (0.846723 × X_1_ × X_2_) − (0.133321 × X_1_ × X_3_) + (1.3429 × X_2_ × X_3_)
L*	= (23.30797 × X_1_) + (28.06543 × X_2_) + (22.59739 × X_3_) − (2.06828 × X_1_ × X_2_) + (26.82039 × X_1_ × X_3_) + (13.26475 × X_2_ × X_3_) − (110.3475 × X_1_ × X_2_ × X_3_) + (364.88044 × X_1_ × X_2_ × (X_1_‐X_2_)^2^) − (365.81906 × X_1_ × X_3_ × (X_1_‐X_3_)^2^) + (410.25535 × X_2_ × X_3_ × (X_2_‐X_3_)^2^)
a*	= (4.94752 × X_1_) + (9.20431 × X_2_) + (5.31943 × X_3_) + (9.50144 × X_1_ × X_3_)
b*	= (−3.35073 × X_1_) + (4.33219 × X_2_) − (3.93189 × X_3_) − (10.44125 × X_1_ × X_2_) + (20.45459 × X_1_ × X_3_) + (8.97593 × X_2_ × X_3_)
C*	= (6.63.85 × X_1_) + (10.90359 × X_2_) + (6.35901 × X_3_) − (9.54302 × X_1_ × X_2_) + (7.49077 × X_1_ × X_3_) − (1.73966 × X_2_ × X_3_)
*h*°	= (333.60077 × X_1_) + (47.61397 × X_2_) + (349.03868 × X_3_) + (652.56985 × X_1_ × X_2_) − (806.46002 × X_1_ × X_3_) − (605.17327 × X_2_ × X_3_)
Hardness (g)	= (710.8525 × X_1_) + (604.86338 × X_2_) + (933.71117 × X_3_) − (2218.87527 × X_1_ × X_2_) − (1848.2777 × X_1_ × X_3_) − (1830.9596 × X_2_ × X_3_) + (16947.34353 × X_1_ × X_2_ × X_3_)
Springiness (%)	= (0.945076 × X_1_) + (0.899273 × X_2_) + (0.933518 × X_3_) + (0.131512 × X_1_ × X_2_) − (1.17739 × X_1_ × X_3_) + (0.216086 × X_2_ × X_3_)
Cohesiveness (s)	= (0.914417 × X_1_) + (0.859726 × X_2_) + (0.953098 × X_3_) + (0.131779 × X_1_ × X_2_) + (0.869015 × X_2_ × X_3_) + (2.93427 × X_1_ × X_2_ × (X_1_‐X_2_)^2^)
Gumminess (g)	= (676.57452 × X_1_) + (486.49535 × X_2_) + (916.37455 × X_3_) − (1841.45157 × X_1_ × X_2_) − (1829.52163 × X_1_ × X_3_) − (1435.29455 × X_2_ × X_3_) + (15285.63365 × X_1_ × X_2_ × X_3_)
Resilience (%)	= (0.294899 × X_1_) + (0.168047 × X_2_) + (0.51166 × X_3_) − (0.341749 × X_1_ × X_2_) − (0.114107 × X_1_ × X_3_) − (0.783081 × X_2_ × X_3_) + (2.08218 × X_1_ × X_2_ × X_3_)

*Note*: X_1_, sour cherry juice concentrate; X_2_, pomegranate juice concentrate; X_3_, grape juice concentrate. X_1_ + X_2_ + X_3_ = 1.00.

The final density may affect molding and confectioneries’ textural behaviors. The density values of the gummy candies obtained using various fruit juices varied between 1.10 and 1.53 g/mL, while the density values of candy samples obtained using glucose syrup were 1.14 ± 0.05 g/mL (Table 2). The highest density was found in trial 11 (a mixture of 0.171% SJC, 0.502% PJC, and 0.327% GJC), while the lowest density was found in trial 2 (1.10 g/mL), trial 12 (1.16 g/mL), and trial 14 (1.18 g/mL), respectively. It can be stated that the percentage of glucose syrup substitution with fruit juice concentrates has a significant effect on density. Also, a significant model was calculated for the impacts of independent variables on this characteristic (*p* < .05). The type and amount of ingredients, cooking process, mixing, and cooling can influence candy density (Arshad et al., 2022). In addition, the sugar acts as a bulking agent and contributes to the overall mass of the candy. In this context, high sugar content can produce a more concentrated solution, leading to a denser candy. However, low sugar content can lead to less dense candies, which may leave more room for air or other ingredients, resulting in a lighter texture (Burey et al., 2009). It was previously reported that (Pekdogan Goztok et al., 2022) the substitution of glucose syrup with fruit juice concentrates results in density values between 0.44 and 0.66 g/mL in marshmallow samples. In the other study of the author, the density of the gels obtained by using only sugar, water, and 8% gelatin was reported as 1.01 ± 0.01 (Pekdogan Goztok et al., 2022). Piliugina et al. (2019) studied gelatin‐forming properties in marshmallow formulation with solubilized substances and natural colorants. They concluded that the density of the marshmallow is between 0.51 and 0.67 g/mL. Chewy candy density is between 1.0 and 0.9 g/ mL, and aerated jelly candy density is between 0.9 and 0.8 g/ mL (Imeson, 2009). In aerated confectionery products like marshmallows, mousses, and whipped creams, gelatin's amphoteric qualities give the air cell wall the desired mechanical resistance, resulting in foaming qualities with low‐density aerated products. It can be stated from the results that formulations that include fruit juice concentrate instead of regular sugar candies impact the interactions between gelatin and sugar in a candy formulation so that it can affect the density of the candy.

**FIGURE 1:**
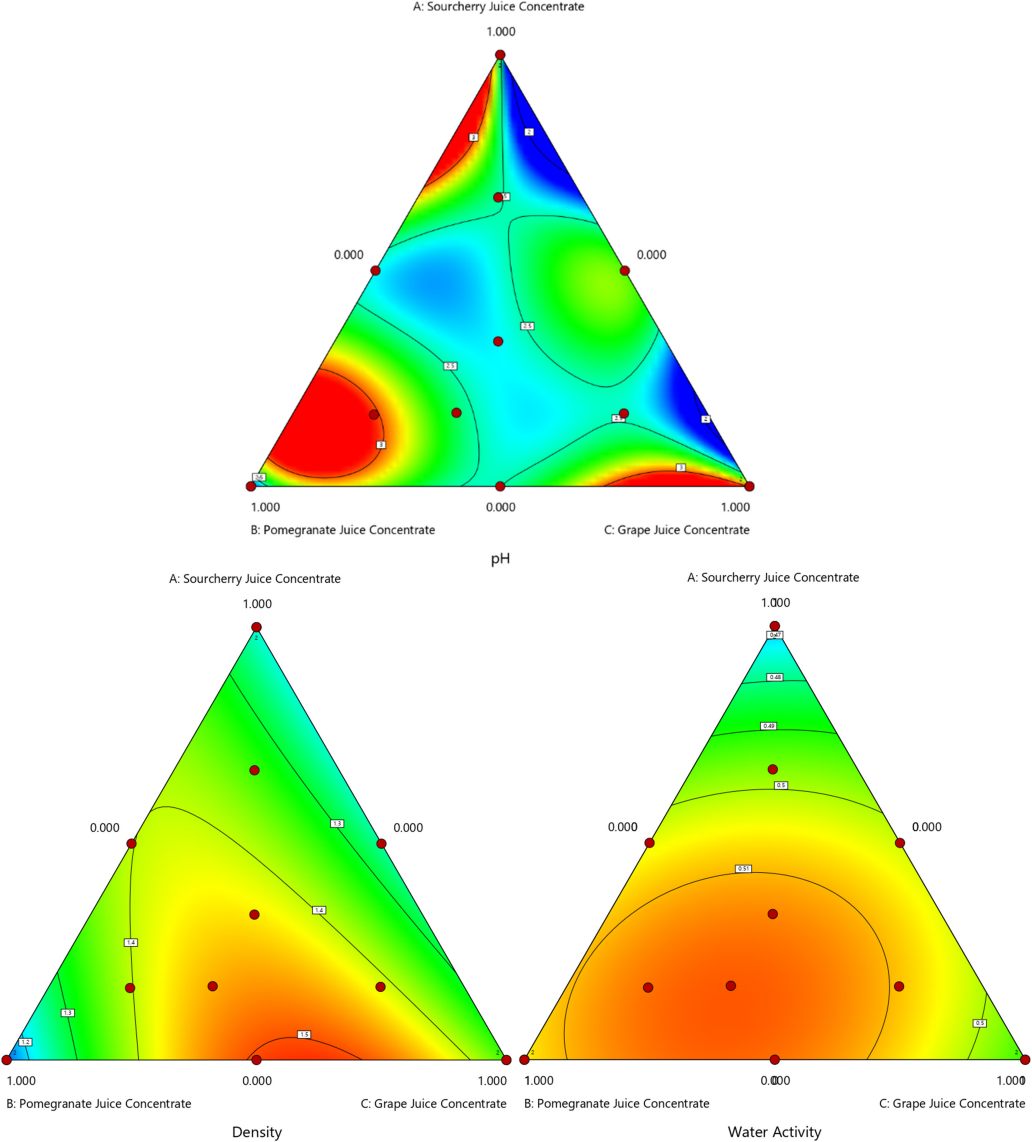
The effect of using different fruit juice concentrates on pH, water activity, and density values of gummy candy.

Water is one of the crucial factors in the processing of confections. It has a distinct effect on texture and shelf life and helps dissolve and prepare sugar and corn (glucose) syrup (Gok et al., 2020). Also, it is stated that no microbial growth occurs below the water activity value of 0.6, and the food is considered safe, so the water activity value of jelly samples during storage is important (Fan et al., 2017; Kowalski et al., 2017). High water content in hard candies may produce a softer stickiness or grainy texture (Ergun et al., 2010). The lowest water activity value was displayed in the candies, where glucose syrup was replaced with SJC. In contrast, the highest water activity value was shown in the candy sample mixture of 0.166% SJC, 0.670% PJC, and 0.164% GJC. The fitted models, depending on the use of sour cherry Juice (X1), pomegranate Juice (X2), and grape Juice Concentrate (X3) were determined as quadratic (Figure 1). The *R* ^2^ value of the quadratic model of water activity results was calculated as 0.8413 (Table 3) and the model equation is given in Table 3. It was seen that the impact of the various fruit juice concentrates on the water activity of candy samples (Table 2) was significant (*p <* .05). Gok et al. (2020) fabricated low‐calorie gummy candy with mannitol and soluble wheat fiber. The water activity of candies was between 0.76 and 0.87. They found a positive correlation between bulking agent level and aw. They concluded that sucrose plays an essential role in water activity by interacting with water molecules and decreasing water activity. Bussiere and Serpelloni (1985) reported that the water activity of gummies and jellies is between 0.5 and 0.75. Nature, the solubility of the ingredient, and its concentration in the confectionary formulations affect water activity (Hartel et al., 2018). In our study, it may be concluded that sugar content could affect water activity; substituting glucose syrup with various fruit juice concentrates may affect the solubility of an ingredient and water activity.

### 3.2 Color properties

**FIGURE 2:**
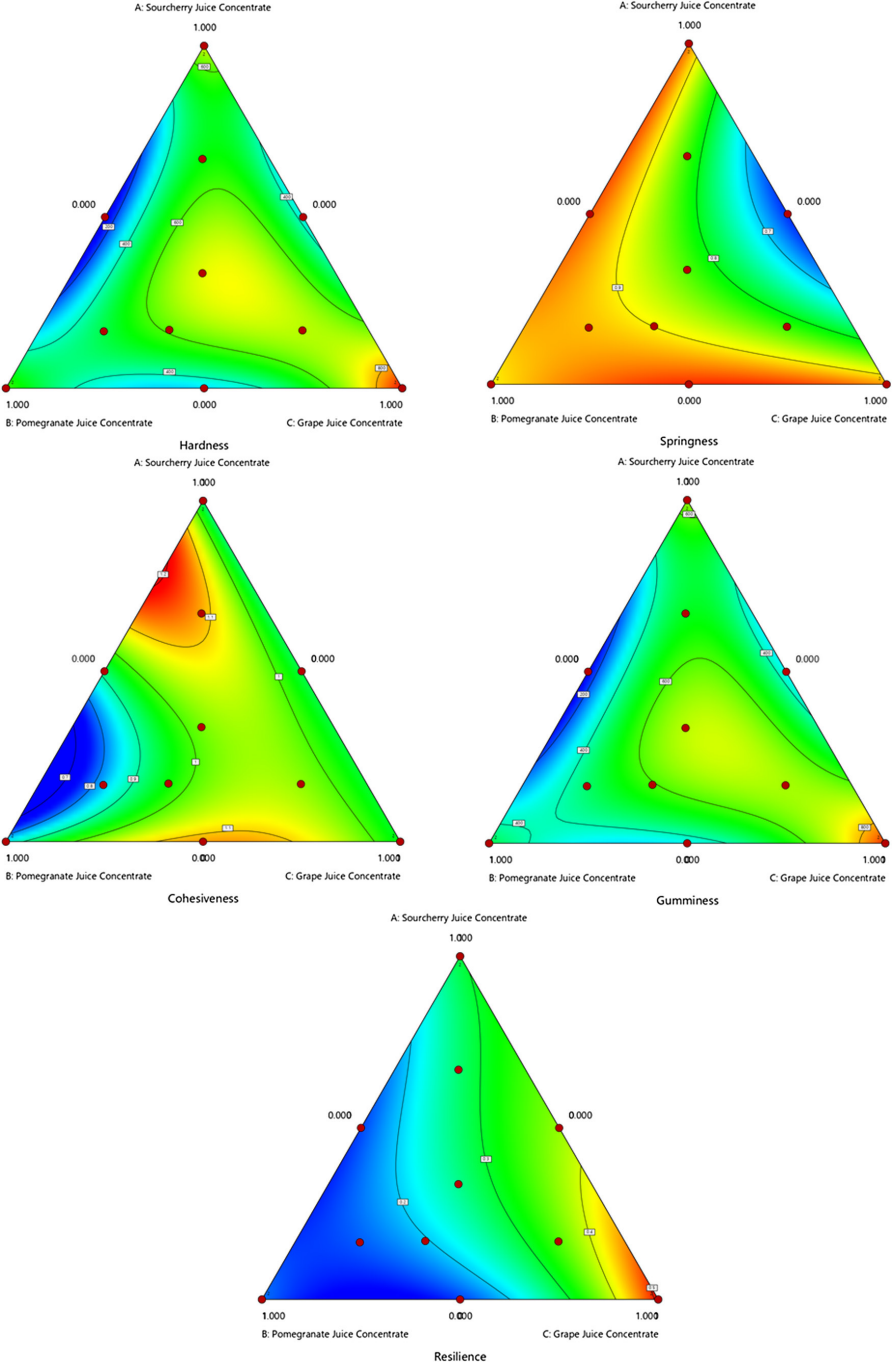
The effect of using different fruit juice concentrates on the color properties of gummy candy.

The color characteristics of gummy candies are important quality parameters due to their high consumer acceptability. All color parameter models, except chroma, were significant (*p* < .05), and the *R* ^2^ values of these models for L*, a*, b*, C*, and *h*° were 0.9375, 0.5792, 0.7126, 0.5640, and 0.6991, respectively (Table 4). The L* and a* models were cubic and R Quadratic cubic, respectively (Table 3). Meanwhile, the other color properties model was quadratic. The results showed that the gummy candies formulated with various fruit juice concentrates had an average redness (a*) value ranging from 4.3 to 11.2. Trial 2 (PJC) obtained a higher reddish color with a* value of (11.2), possibly caused by anthocyanin pigments in pomegranate fruit juice (Figure 2). Anthocyanins and copigments have a significant influence on color intensity (Ventura et al., 2013). According to legal regulations, using natural source colorants for confectionery products is necessary from a health point of view. Recently, researchers have focused on using fruit and fruit‐derived pigments to develop a natural colorant in confectionery products to eliminate the potential risks of synthetic colorants. Due to the rise of attention‐deficit hyperactivity disorder (ADHD), which is associated with increased consumption of artificial food colors, there are many studies utilizing natural colors, especially in most preferred products like candies (Cano‐Lamadrid et al., 2020; Moghaddas Kia et al., 2020; Nishiyama‐Hortense et al., 2022; Pekdogan Goztok et al., 2022). A previous study of pomegranate‐based jellies concluded that the gelatin dose and sweetener significantly affected the color parameters of jelly candies (Cano‐Lamadrid et al., 2020) as the primary influencing factors for liking jellies were their high reddish hue and brightness. High sugar content promotes the Maillard reaction, thus altering the color of the jellies (Anjliany et al., 2022). Color characteristics of the gummy candy were strongly influenced by temperature. The main obstacle to utilizing natural colorants is their susceptibility to high temperatures (Charoen et al., 2015; Moghaddas Kia et al., 2020).

**TABLE 4:** Color properties and fitted model statistics of gummy candies.

Sample	L*	a*	b*	Chroma	°*h*
1	24.4 ± 1.90	6.58 ± 1.63	−1.72 ± 1.51	6.99 ± 1.05	343.6 ± 17.2
2	28.9 ± 0.81	11.2 ± 0.48	6.28 ± 1.06	12.8 ± 0.90	29.3 ± 3.24
3	25.5 ± 3.42	5.49 ± 0.19	−2.59 ± 0.82	6.10 ± 0.46	335.0 ± 6.53
4	22.4 ± 1.67	5.35 ± 0.16	−3.43 ± 0.78	6.38 ± 0.38	327.6 ± 6.19
5	28.9 ± 0.73	7.40 ± 0.55	1.96 ± 2.12	7.83 ± 0.88	23.4 ± 1.65
6	29.9 ± 0.85	8.80 ± 0.52	3.10 ± 0.90	9.35 ± 0.77	19.2 ± 4.11
7	24.9 ± 0.18	6.15 ± 1.39	−1.67 ± 1.48	6.55 ± 0.88	343.4 ± 16.5
8	22.9 ± 0.51	5.67 ± 0.27	−2.67 ± 0.05	6.26 ± 0.23	334.8 ± 1.38
9	27.7 ± 0.36	9.62 ± 1.03	4.31 ± 1.03	10.6 ± 1.33	23.9 ± 3.22
10	25.8 ± 3.20	4.65 ± 0.88	−1.65 ± 2.32	5.12 ± 1.56	343.98 ± 22.54
11	30.7 ± 2.35	7.40 ± 1.10	4.58 ± 3.86	9.11 ± 2.75	28.5 ± 18.20
12	28.38 ± 4.14	8.47 ± 0.74	2.49 ± 0.33	8.83 ± 0.62	16.50 ± 3.40
13	22.9 ± 0.66	5.79 ± 0.11	−3.46 ± 0.01	6.75 ± 0.09	329.1 ± 0.44
14	22.3 ± 2.24	4.30 ± 0.54	−4.79 ± 0.54	6.44 ± 0.76	311.9 ± 0.42
Model	Cubic	R Quadratic	Quadratic	Quadratic	Quadratic
*R* ^2^	0.9375	0.5792	0.7126	0.5640	0.6991
Adjusted‐*R* ^2^	0.7969	0.4529	0.5330	0.2914	0.5110
Predicted‐*R* ^2^	−9.2026	−0.0435	0.0642	−0.4204	−0.2208
Adeq, precision	7.2126	5.7633	5.2598	4.0916	4.5414
*F*‐value	6.67	4.59	3.97	2.07	3.72
*p*‐value	0.0417*	0.0288*	0.0417*	0.1722**	0.0490*
Lack of fit	0.1797**	0.7546**	0.5375**	0.6973**	0.0503**

*Note*: *p* < .05, R: reduced, L*: brightness, a*: ±red‐green, and b*: ±yellow‐blue, C: chroma, *h*°: hue angle, mean ± SD, five replicates of the color (L*; brightness, C*; chroma, and *h*°; hue angle) experiments were performed.

* Significant.

** Not significant.

### 3.3 Textural properties

**FIGURE 3:**
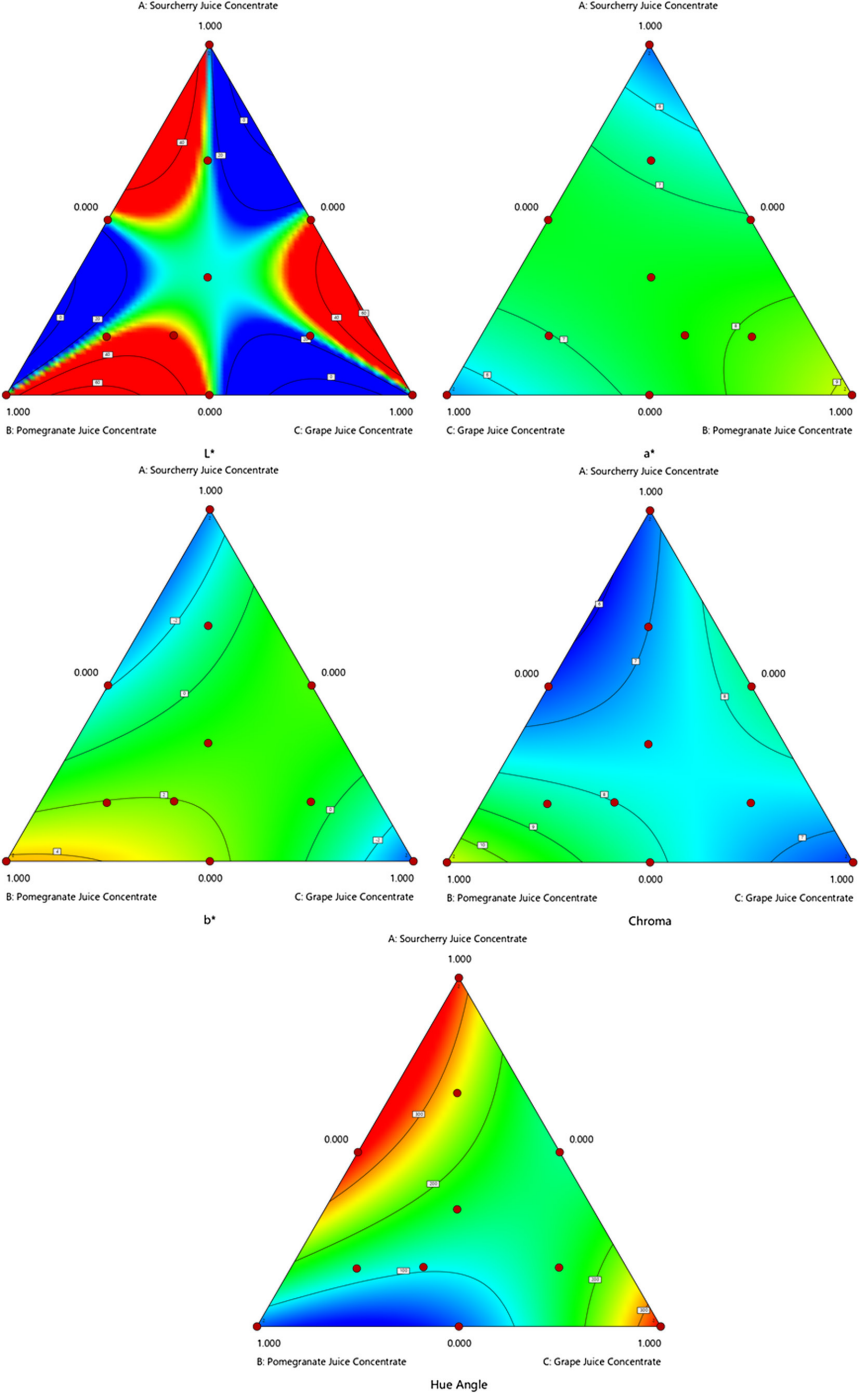
The effect of using different fruit juice concentrates on the textural properties of gummy candy.

The sensory properties of confectionery are related to their textural parameters. Therefore, textural properties have been investigated as important quality criteria in confectionery products such as candies (Gok et al., 2020; Hartel et al., 2018; Pekdogan Goztok et al., 2022). Hardness is one of the crucial factors in producing candy because it affects the quality and consumer perception as well as determines the strength of the gel network (Altınok et al., 2020). The effect of substituting glucose syrup with various fruit juice concentrates on the texture characteristics of the candies is shown in Table 5. The hardness (146.1–938.8 g), springiness (0.63–0.99%), cohesiveness (0.75–1.19 s), gumminess (136.02–947.94 g), and resilience (0.12–0.51%) values of gummy candies were determined. The hardness, springiness, cohesiveness, gumminess, and resilience values of the candies obtained using glucose syrup were 2809.3 g, 0.74%, 0.78 s, 2189 g, and 0.11%, respectively. The texture properties of candies were influenced by fruit juice concentration instead of glucose syrup (Figure 3). The fruit juice concentrates in the formulation caused a decrease in the hardness and gumminess values of the candies, and the softer candies were obtained. Significant hardness, gumminess, and resilience models were calculated (*p <* .05) with *R* ^2^ values of 0.7856, 0.7794, and 0.9729, respectively. The gelatin concentration and strength, glucose syrup‐to‐sucrose ratio, sugar type, sugar content, and sugar‐to‐gelatin ratio can influence the texture of gummy candy (Altınok et al., 2020; Kurt et al., 2022; Pekdogan Goztok et al., 2022; Tireki et al., 2021). Kurt et al. (2022) and Mutlu et al. (2018) concluded that hardness depends on gelatin concentration and a higher gelatin dose results in a rigid gel. In another study, Tireki et al. (2021) found that glucose syrup:sucrose ratio and gelatin concentration significantly impacted the hardness of gummy confections. This was confirmed by our study, where we found that the differences in texture between samples could be attributed to the various sugar contents of fruit concentrates. Sugars promote the network structures of gelatin gels and enhance their gel strength. In addition, substituting glucose syrup with various fruit juice concentrates may affect the binding ability of water molecules and the crystallization behavior of the candies.

**TABLE 5:** Texture properties and fitted model statistics of gummy candies.

Sample	Hardness (g)	Springiness (%)	Cohesiveness (s)	Gumminess (g)	Resilience (%)
1	741.0 ± 160.7	0.94 ± 0.04	0.91 ± 0.02	673.16 ± 149.3	0.28 ± 0.01
2	807.0 ± 328.2	0.83 ± 0.11	0.75 ± 0.05	602.14 ± 239.9	0.21 ± 0.03
3	146.1 ± 58.9	0.99 ± 0.01	0.94 ± 0.05	136.02 ± 53.2	0.15 ± 0.02
4	863.0 ± 136.8	0.94 ± 0.04	0.96 ± 0.01	832.08 ± 134.8	0.51 ± 0.02
5	258.3 ± 96.73	0.96 ± 0.01	1.17 ± 0.11	301.30 ± 119.3	0.15 ± 0.01
6	297.7 ± 62.3	0.63 ± 0.11	0.87 ± 0.05	258.63 ± 60.0	0.38 ± 0.04
7	575.3 ± 221.8	0.87 ± 0.04	1.19 ± 0.10	671.36 ± 215.5	0.26 ± 0.02
8	919.6 ± 462.8	0.92 ± 0.02	0.99 ± 0.06	884.79 ± 379.8	0.35 ± 0.02
9	376.8 ± 109.4	0.86 ± 0.13	0.88 ± 0.08	338.13 ± 123.6	0.18 ± 0.03
10	566.7 ± 167.3	0.72 ± 0.04	0.84 ± 0.03	476.77 ± 153.3	0.28 ± 0.04
11	704.6 ± 162.7	0.96 ± 0.02	1.01 ± 0.10	708.00 ± 156.3	0.17 ± 0.02
12	441.0 ± 154.2	0.98 ± 0.00	0.94 ± 0.14	414.45 ± 162.1	0.12 ± 0.02
13	938.8 ± 120.0	0.90 ± 0.05	1.01 ± 0.03	947.94 ± 118.5	0.51 ± 0.04
14	679.7 ± 225.1	0.94 ± 0.05	0.95 ± 0.03	645.11 ± 215.5	0.31 ± 0.02
Model	S Cubic	Quadratic	Quadratic	S Cubic	S Cubic
*R* ^2^	0.7856	0.6763	0.5744	0.7794	0.9729
Adjusted‐*R* ^2^	0.6018	0.4740	0.3083	0.5903	0.9497
Predicted‐*R* ^2^	−0.5332	0.2036	−0.2528	−0.5339	0.9052
Adeq, Precision	7.2518	6.6809	4.5876	7.0787	18.1963
*F*‐value	4.27	3.34		4.12	41.88
*p*‐value	0.0393*	0.0633**		0.0429*	<0.0001*
Lack of fit	0.4671**	0.3424**		0.1198**	0.9166**

*Note*: *p* < .05. Mean ± SD. S, special. Five replicates of texture profile analysis were performed.

* Significant.

** Not significant.

Furthermore, it was determined that the hardness and gumminess values were lower in the mixtures (trials 3, 5, and 6), in which 50% of the fruit juice concentrates were used compared to the samples in which 100% rates (trials 1, 2, and 4 and 12–14) were used. The influence of the type and amount of fruit juice used on the texture characteristics depends on the polyphenol content and derivative compounds in the fruit juice concentrate. Phenolic compounds interact noncovalently with gelatin, which has a protein structure (Le Bourvellec & Renard, 2012; Ozdal et al., 2013). The total phenolic matter content of the fruit juice concentrates used in this study is in the form of SJC > PJC > GJC (Mahdavi et al., 2011). When the hardness value of the fruit juice concentrates used in 100% ratios is considered, SJC < PJC < GJC. Since phenolic compounds affect the water‐binding ability of gelatin, it is suggested to increase the gelatin and/or bloom value in formulation with high polyphenol component content (Schrieber & Gareis, 2007). In our study, utilizing different concentrations of fruit juice and polyphenol component content as a potential replacement for corn syrup affects the gelling power of gelatin. In addition, the presence of sugar may enhance the interaction between gelatin molecules through the reduction of water activity (Arshad et al., 2022). In this regard, Pekdogan Goztok et al. (2022) concluded that differences in texture properties may be attributed to various glucose/fructose ratios and types of sugar (mono‐ and di‐saccharides) in fruit juices.

### 3.4 Microstructure of gummy candies

**FIGURE 4:**
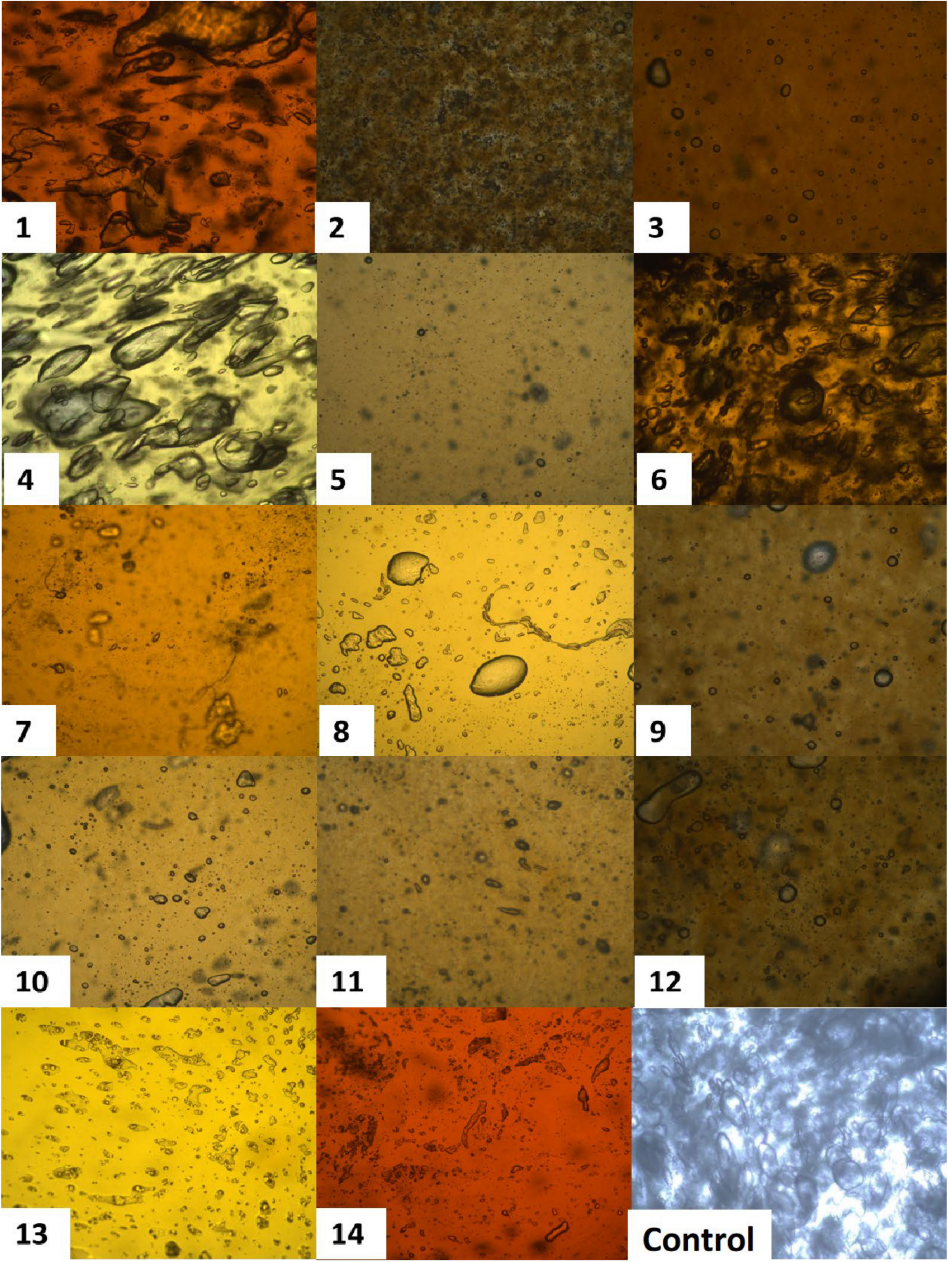
Microstructures of gummy candy produced with different fruit juice concentrates.

The microstructures of the candy samples examined by polarized light microscopy in this study are given in Figure 4. As a result, the microstructural properties of the samples are generally heterogeneous and have different porous sizes. It was observed that the pore sizes of the samples in trial 1 (100% SJC) and trial 4 (100% GJC) were larger than those of the PJC samples. In the mixture of concentrates of fruit juices, a decrease in the pore size of the candies in general was determined, except for the samples with SJC and GJC (50% w/w) mixture. The amount of polymers in the gels, pore size, and structure affect the water retention/viscosity of the gels (Mohammadian & Madadlou, 2016). Phenolic compounds and sugar profiles in different fruit juices used in this study were found to be effective on microstructure.

## 4 CONCLUSION

The D‐optimal mixture design approach was used to optimize the formulation of gummy candy with various fruit juice concentrates. This study demonstrated that fruit juice concentrates (sour cherry, pomegranate, and grape juice concentrates) could be a potential substitute for gummy candy production to meet consumers’ demands and expectations of healthier confectionery products. Water activity, soluble solid content, texture properties like hardness, springiness, cohesiveness, resilience, and color properties of gummy candy were modeled. The gummy candies’ water activity values varied in the range of 0.46–0.52. The pH values and density were in the range of 2.22–3.08 and 1.10–1.53 g/mL, respectively. The quadratic model expressing the effect of the fruit juice concentration mixture on the pH, aw, and density values was calculated as significant (*p <* .05), and the *R* ^2^ values of these models were found to be 0.9401, 0.8413, and 0.8533, respectively. In addition, the color characteristics (L*, a*, b*, and *h*°) of the products were significant (*p <* .05) by using various fruit juice concentrate, and *R* ^2^ values of these models were 0.9375, 0.5792, 0.7126, and 0.6991, respectively. However, model statistics showed that the choice of juice concentration for glucose syrup replacement was not significant (*p >* .05) regarding springiness. In contrast, the models were significant (*p <* .05) for hardness, gumminess, and resilience with *R* ^2^ values of 0.7856, 0.7794, and 0.9729, respectively. Phenolic compounds and mono‐ and di‐saccharide differences in fruit juices might be effective on gummy candy's texture and microstructure properties. It has been demonstrated that applying these fruit juice concentrates in gummy candy formulation makes it possible to fabricate alternative products with higher health‐beneficial effects. Therefore, it is essential to determine the sensory properties to achieve consumer acceptance potential with future research on this topic.

## AUTHOR CONTRIBUTIONS

Omer Said Toker: Conceptualization, writing—review & editing. Ibrahim Palabiyik: Conceptualization, writing—review & editing. Nevzat Konar: Conceptualization, writing—review & editing. Serpil Pekdogan Goztok: Methodology, formal analysis. Arezou Habibzadeh Khiabani: Writing—review & editing.

## CONFLICT OF INTEREST STATEMENT

This article's authors declare that they have no competing financial interests or personal relationships that might have influenced the findings.

## ETHICS STATEMENT

Ethics approval was not required for this research.

## Data Availability

Data sharing is not applicable to this article as all data generated or analyzed during this study are included in this published article and its supporting information files.
